# IL-13 Alleviates Cardiomyocyte Apoptosis by Improving Fatty Acid Oxidation in Mitochondria

**DOI:** 10.3389/fcell.2021.736603

**Published:** 2021-09-17

**Authors:** Xiaoyu Guo, Ting Hong, Shen Zhang, Yazhong Wei, Haizhen Jin, Qing Miao, Kai Wang, Miao Zhou, Chong Wang, Bin He

**Affiliations:** ^1^Department of Critical Care Medicine, Shanghai Chest Hospital, Shanghai Jiao Tong University, Shanghai, China; ^2^Central Laboratory of Shanghai Chest Hospital, Shanghai Jiao Tong University, Shanghai, China; ^3^Department of Anesthesiology, Shanghai Chest Hospital, Shanghai Jiao Tong University, Shanghai, China; ^4^Department of Anesthesiology and Intensive Care Medicine, Xinhua Hospital, School of Medicine, Shanghai Jiao Tong University, Shanghai, China; ^5^Department of Cardiovascular Surgery, Shanghai Chest Hospital, Shanghai Jiao Tong University, Shanghai, China

**Keywords:** sepsis, cardiomyocyte apoptosis, IL-13, mitochondria, fatty acid

## Abstract

Sepsis-induced cardiac injury (*SIC*) is one of the most common complications in the intensive care unit (ICU) with high morbidity and mortality. Mitochondrial dysfunction is one of the main reasons for *SIC*, and Interleukin-13 (IL-13) is a master regulator of mitochondria biogenesis. The aim of the present study was to investigate the role of IL-13 in *SIC* and explore the underlying mechanism. It was found that reactive oxygen species (ROS) production and apoptosis were significantly increased in lipopolysaccharide (LPS)-stimulated primary cardiomyocytes, which was accompanied with obvious mitochondria dysfunction. The results of RNA-sequencing (RNA-seq), mitochondrial membrane potential, fatty acid uptake and oxidation rate suggested that treatment with IL-13 could restore the function and morphology of mitochondria, indicating that it played an important role in protecting septic cardiomyocytes. These findings demonstrated that IL-13 alleviated sepsis-induced cardiac inflammation and apoptosis by improving mitochondrial fatty acid uptake and oxidation, suggesting that IL-13 may prove to be a potential promising target for *SIC* treatment.

## Introduction

Sepsis-induced myocardial dysfunction is one of the main causes of death in the intensive care unit (ICU). Various theories and therapies have been proposed to treat septic patients with impaired cardiac function (Ichinose et al., 2007; Xu et al., 2012; Venet and Monneret, 2018). In recent years, mitochondrial dysfunction has been considered as a crucial cause of sepsis-induced cardiac injury (*SIC*) with augmented release of reactive oxygen species (ROS) and decreased mitochondrial oxidative phosphorylation (Drosatos et al., 2011, 2013; Schilling et al., 2011). Toxic ROS and insufficient energy metabolism finally lead to the impairment and apoptosis of cardiomyocytes (Sepúlveda et al., 2020).

Interleukin-13 (IL-13) is a protein secreted by many cell types and recognized as a type 2 immunity cytokine that plays an important role in a variety of diseases, including allergic inflammation, schistosomiasis, and tissue repair (Qian et al., 2021). Recently, IL-13 is reported to be increased after endurance exercise, probably due to type 2 innate lymphocytes (ILC2) expansion in the muscle. IL-13 preserves the ability of fatty acid utilization and mitochondrial biogenesis in the muscle (Knudsen et al., 2020). Although IL-13 is known to have a protective effect in various diseases, whether IL-13 also has a cardioprotective effect in sepsis remains to be defined.

In this study, we investigated the potential role of IL-13 in protecting mitochondria and alleviating *SIC*. IL-13 decreased the apoptosis and increased the cardiac function in *SIC*. Furthermore, we evaluated the morphology of mitochondria and expression level of ROS in different conditions. Finally, we explored the possible mechanisms whereby IL-13 recovered the fatty acid utilization of mitochondria in the septic state. Our findings suggest that IL-13 may be a promising therapeutic target in *SIC*.

## Materials and Methods

### Primary Cardiomyocyte Culture

Primary cardiomyocytes were isolated from neonatal rats aged 1–3 days (Wang et al., 2020). Briefly, the neonatal SD rat heart was digested into single cells in 0.1% trypsin (Gibco) diluted with Hank’s balanced salt solution (HBSS, Cytiva) at 37°C. Then, low glucose Dulbecco’s Modified Eagle’s Medium (DMEM, Cytiva) containing fetal bovine serum (FBS) was added into the single cardiomyocyte suspension to neutralize the trypsin. In the end, the cardiomyocytes were cultured in low glucose DMEM containing 10% FBS at 37°C with 5% CO_2_.

### Model Establishment

The cellular model of *SIC* was established by lipopolysaccharide (LPS, Sigma-Aldrich, United States) incubation. Briefly, primary cardiomyocytes were cultured in low glucose DMEM containing 10 ug/ml LPS for 6 h. Morphology and pulse rhythm of cardiomyocytes were observed.

C57BL/6 mice (JSJ Co., Shanghai, China) were raised in the specific pathogen free (SPF)-grade environment, all biosecurity as well as institutional safety procedures were approved and supervised by the Ethics Committee of Shanghai Chest Hospital (Shanghai, China). C57BL/6 were intraperitoneally (i.p.) injected with LPS at a dosage of 10 mg/ml for 6 h.

### Adenosine Triphosphate Measurement

Adenosine Triphosphate (ATP) production was measured by ATP measurement assay (Beyotime, China) according to the operating manual. Briefly, lysis solution was added into the cardiomyocytes and then centrifuged at 12000 × *g* at 4°C for 5 min. Then, the ATP standard solution was diluted to an appropriate concentration gradient with ATP test solution, and 100 ul ATP test solution was added into the microplate for 3 min in advance. Finally, 20 ul standard solution and sample solution were added into the prepared ATP test solution and detected by luminometry (Thermo Fisher Scientific, United States).

### Reactive Oxygen Species Measurement

Generation of ROS was detected with ROS Assay Kit (Beyotime, China). In brief, DCFH-DA was diluted to a concentration of 10 uM. Cardiomyocytes were washed with PBS firstly and incubated with DCFH-DA at 37°C for 20 min. After that, cells were washed with PBS for 3 times and detected by fluorescent microscopy.

### Mitochondrial Morphology

Mitochondria of primary cardiomyocytes were stained with MitoTracker^TM^ Deep Red (Invitrogen, United States). Briefly, 1 mM mitoTracker solution was diluted to the final work concentration of 50 nM with DMEM. Primary cardiomyocytes were washed with PBS and incubated with mitoTracker work solution for 30 min at 37°C. After staining, cells were washed with PBS for 3 times again and added fresh DMEM.

### TUNEL Staining

TUNEL assays were performed with sections using One-step TUNEL Cell Apoptosis Detection Kit (Beyotime, China) principally according to the supplier’s instruction. In addition, the nuclei in the slices were labeled with DAPI, and finally photographed with a fluorescence microscope.

### Isolation of Mitochondria

Mitochondria from primary cardiomyocytes were extracted according to the instructions of QIAGEN (United States). Cells were collected and added with lysis buffer and incubated at 4°C for 10 min. After that, samples were centrifuged at 1000 × *g* at 4°C for 10 min. The precipitation was collected, incubated with disruption buffer, and then homogenized using a Dounce tissue grinder. The supernatant was collected in a new clear tube after 1000 × *g* centrifuge at 4°C for 10 min. Next, the samples were centrifuged again at 6000 × *g* for 10 min and added with 1 ml mitochondria, stored, and suspended until precipitation. Finally, the samples were centrifuged at 6000 × *g* for 20 min, resuspended and stored for future use.

### Extraction and qPCR of Mitochondria DNA

The gene expression of mitochondria DNA (mtDNA) was determined by RT-qPCR with SYBR Green. Relative expression was quantified to Rplp0 as the internal standard control. All primer sequences are listed in Supplementary Table 1.

### Western Blot Analysis

Protein lysates and Western blotting were performed using the classic methods. Briefly, each protein sample (30 ug) was resuspended in SDS-PAGE loading buffer, boiled at 95°C for 10 min and electrophoresed with appropriate gels. After that, the protein was electrotransferred to PVDF membrane (Millipore, United States) and blocked with 5% non-fat milk for 1 h at room temperature. Antibodies of Bcl2, Bax, Caspase3, α-tubulin, α-actin and Cox4 (Cell Signaling Technology, United States) and OXPHOS (Invitrogen, United States) were added to bind the membrane overnight at 4°C. Then, the membrane was washed with TBST for 3 times and incubated with HRP-conjugated secondary antibodies (Cell Signaling Technology, United States) for 1 h at room temperature. Finally, the PVDF membrane was washed with TBST for 3 times and developed with electrochemiluminescence solution (Millipore, United States).

### Transmission Electron Microscope Investigation

The heart was isolated from the LPS-induced cardiac injury mice and cut into 1 mm^3^ sections and incubated with fixative solution (Daixuan Biotechnology Co., Ltd., China) immediately, followed by incubation with 2.5% glutaraldehyde overnight. After that, the fixed samples were washed by ddH_2_O and put into 30, 50 and 70% ethanol at 4°C for 10 min each in sequence. Then it was given to 80, 90 and 95% acetone for 10 min one by one, and to 100% acetone for 10 min × 2. Afterward, the specimen was immersed into epoxy resin and shaken at 30°C for 4 h. Finally, the prepared sample was sliced to ultrathin sections for transmission electron microscope (TEM) observation (Hitachi, Tokyo, Japan).

### Mitochondrial Membrane Potential Detection

Mitochondrial membrane potential was detected with JC-10 Mitochondrial Membrane Potential Assay Kit (Yeasen, China). According to the instructions, JC-10 stock solution was diluted with ddH_2_O at the ratio of 1:1000 to make JC-10 work solution. Primary cardiomyocytes were washed with PBS and incubated with 1 ml DMEM mixed with 1 ml JC-10 work solution at 37°C for 20 min. After that, cell samples were washed with PBS twice and added with fresh DMEM for confocal laser microscopic observation.

### RNA Sequencing and Analysis

RNA was extracted from heart tissues that were grown under the appropriate conditions using the RNeasy mini kit (Qiagen, United States). After that, RNA-sequencing (RNA-seq) was performed by GENEWIZ (Suzhou, China). Genes with a | fold-change (FC) | > 1 and *P* < 0.05 were defined as significant differential expressed genes for further analysis.

### Fatty Acid Uptake Rate Detection

According to the instruction book, primary cardiomyocytes were seeded into black 96-well plate and incubated with LPS or IL-13. After that, 100 ul fatty acid dye solution (Sigma-Aldrich, United States) was added for 1 h incubation. Finally, fluorescence was detected with VARIOSKAN LUX (Thermo Fisher Scientific, United States) and reported as Fluorescence (RFU).

### Fatty Acid Oxidation Rate Detection

Fatty acid oxidation rate assay (Genmed Scientifics Inc., United States) was applied to detect the rate of β-oxidation rate. According to the instruction manuscript, primary cardiomyocytes were seeded into 96-well plate. After stimulation, substrate of palmitoyl carnitine was added into the 96-well plates for β-oxidation. Finally, VARIOSKAN LUX was used to detect OD values at 420 nm. Fatty acid β-oxidation rate = [(ODsample-ODbackgroud) × system volume (ml)]/[sample protein content (mg) × 105 × reaction time(min)].

### Statistical Analysis

Data are presented as the mean ± SEM at least three duplications of different samples. Student’s *t*-test was applied for analysis between two groups and one-way analysis of variance (ANOVA) was used for comparisons between multiple groups. GraphPad Prism 7.0 (GraphPad Software Inc., United States) were used to analyze and illustrate the data. Differences with *p*-values < 0.05 were considered statistically significant.

## Results

### Interleukin-13 Alleviates LPS-Induced Cardiomyocyte Apoptosis and ROS Production *in vivo*

**FIGURE 1:**
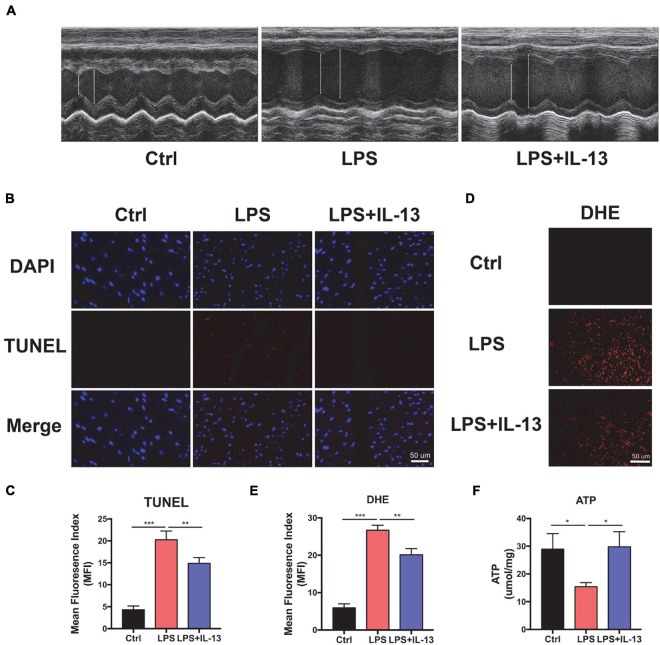
Interleukin-13 (IL-13) alleviates cardiomyocyte apoptosis and reactive oxygen species (ROS) production *in vivo*. **(A)** Representative figures of echocardiography (ECG). **(B)** TUNEL staining was used to observe the level of apoptosis and **(C)** quantitative results were calculated with image J. **(D)** The level of ROS was observed with DHE probe and **(E)** calculated. **(F)** The level of Adenosine Triphosphate (ATP) production. Data are shown as the mean ± SEM (*n* = 3). *P < 0.05; **P < 0.01; ***P < 0.001.

To understand the potential role of IL-13 in protection against *SIC*, LPS was injected i.p. to induce a cytokine storm. Echocardiography (ECG) was employed to determine the cardiac function in LPS-induced cardiac injury. As shown in Figure 1A, myocardial systolic function was decreased after LPS injection, and IL-13 mitigated cardiac injury and reversed to a certain extent. Eject fraction (EF) and fraction shorting (FS) were calculated in Supplementary Figures 1A,B. The heart was then harvested and TUNEL stained. As shown in Figures 1B,C, compared with the control group, LPS induced obvious cardiomyocyte apoptosis and IL-13 attenuated the apoptosis. In addition, DHE staining was employed to evaluate the ROS production in LPS-induced cardiac injury. As shown in Figures 1D,E, the change of ROS production was in accordance with the level of apoptosis, suggesting that ROS may be an important cause of cardiomyocyte apoptosis. Furthermore, H&E staining showed that LPS caused edema of the heart tissue as represented by enlarged tissue space (Supplementary Figure 2). We also observed the change of ATP in the heart of the septic mice, which is a crucial form of energy for the heart. Interestingly, LPS decreased the production of ATP and IL-13 treatment eliminated the disorder of energy metabolism (Figure 1F).

### Interleukin-13 Alleviates LPS-Induced Cardiomyocyte Apoptosis and ROS Production *in vitro*

**FIGURE 2:**
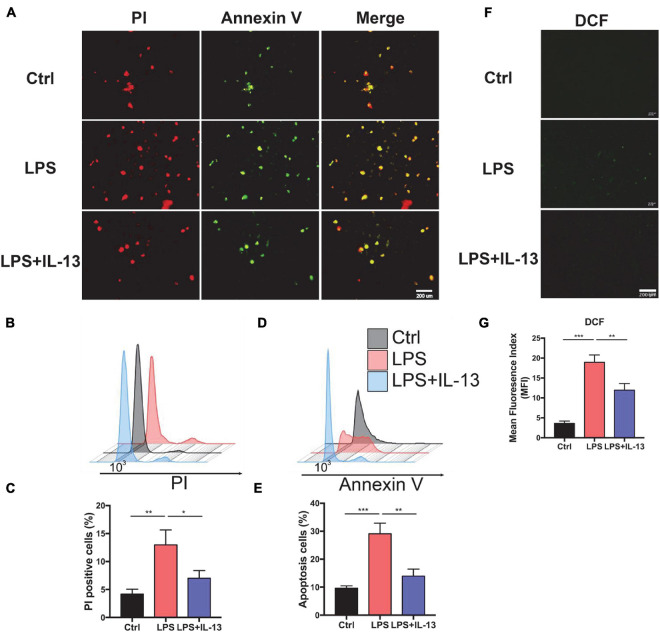
Interleukin-13 alleviates cardiomyocyte apoptosis and ROS production *in vitro*. **(A)** PI and Annexin V dye was used to observe the level of apoptosis. **(B)** PI positive cells were counted by flow cytometry and **(C)** calculated. Annexin V positive cells **(D)** and the apoptosis ratio **(E)** were counted by flow cytometry and calculated. **(F)** DCFH-DA staining was applied to detect the level of ROS and **(G)** measured with image J. Data are shown as the mean ± SEM (*n* = 3). *P < 0.05; **P < 0.01; ***P < 0.001.

Next, we explored the therapeutic effect of IL-13 with primary cardiomyocytes. PBS, 10 ug/ml LPS and LPS + IL-13(50 ng/ml) was added to cardiomyocytes, respectively. After that, PI and Annexin V dyes were employed to mark the necrotic and apoptotic cells, respectively. As shown in Figure 2A, necrotic and apoptotic cells were increased conspicuously in LPS-stimulated group as compared with the control group, while IL-13 relieved the necrosis and apoptosis effectively. To obtain a quantification result of apoptosis, we used flow cytometry for more accurate investigation. As shown in Figures 2B,C, PI positive cells were decreased markedly in IL-13 treatment group as compared with LPS group (13.6% *vs.* 5.6%). Meanwhile, Annexin V positive cells, which represent the total apoptotic cells, were also decreased after IL-13 treatment (Figures 2D,E). In addition, we also detected the ROS production using the DCFH-DA assay kit. As shown in Figures 2F–G, the level of ROS in normal primary cardiomyocytes was very low but increased markedly after LPS induction. Together, we conclude that IL-13 could protect the cardiomyocytes and reduce the production of ROS.

### Interleukin-13 Sustains the Homeostasis of Mitochondria

**FIGURE 3:**
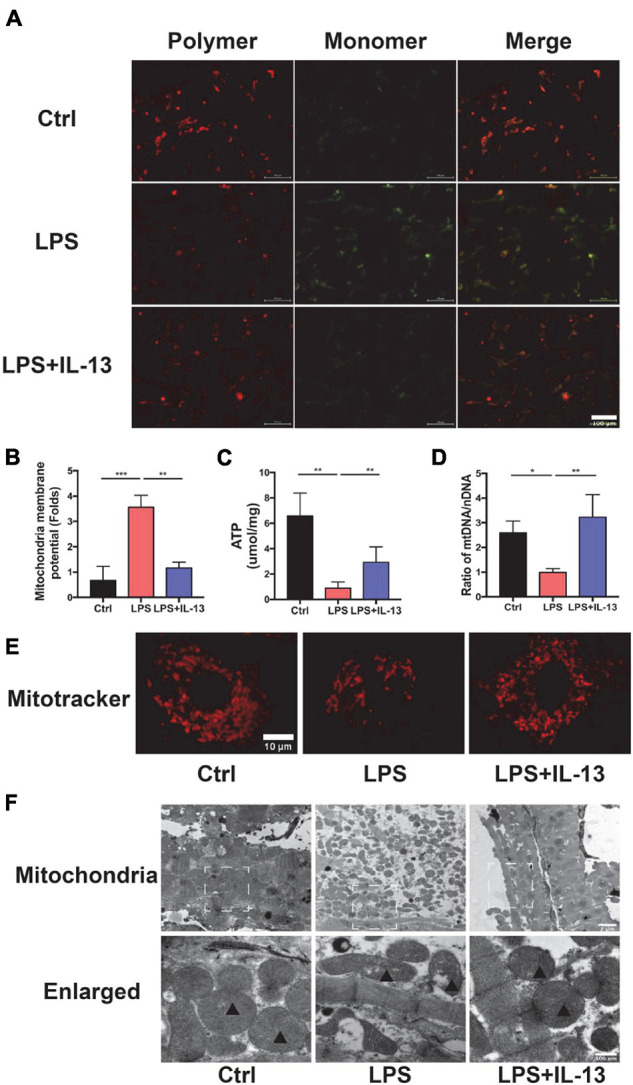
Interleukin-13 recovers homeostasis and morphology of mitochondria. **(A)** Measurement of mitochondrial membrane potential using the JC-10 probe. **(B)** Calculation of the membrane potential change ratio with a monomer/polymer. **(C)** Level of ATP production. **(D)** mitochondria DNA (mtDNA)/nuclear DNA (nDNA) ratio. **(E)** Mitochondrial morphology shown by MitoTracker deep red staining. **(F)** Mitochondrial morphology shown by transmission electron microscope (TEM). Mitochondria was labeled with black triangle. Data are expressed as the mean ± SEM (*n* = 3). *P < 0.05; **P < 0.01; ***P < 0.001.

The above results suggest that IL-13 could alleviate the apoptosis of cardiomyocytes but the exact mechanism remains to be further understood. In consideration of the change in ATP production, IL-13 may play a protective role by maintaining the homeostasis of mitochondria. Next, we evaluated the mitochondria function by detecting the membrane potential by JC-10, which is a bicolourable membrane potential probe that reflects the function of mitochondria. JC-10 presents the polymer that displays red fluorescence in mitochondria of normal cardiomyocytes but resolves into a monomer with green fluorescence when damage occurs in mitochondria. As shown in Figures 3A,B, red fluorescence decreased, and green fluorescence increased after LPS stimulation. IL-13 recovered the membrane potential of mitochondria by regulating its homeostasis. After that, we detected the production of ATP in primary cardiomyocytes. As shown in Figure 3C, IL-13 increased ATP production as expected. In addition, IL-13 treatment increased the biogenesis of mitochondria as determined by the ratio of mtDNA and nuclear DNA (nDNA; Figure 3D).

### Interleukin-13 Sustains the Morphology of Mitochondria

In addition to the functional change of mitochondria, the morphology also restored to normal after IL-13 treatment. A deep red MitoTracker dye was applied to evaluate the change of mitochondria. As shown in Figure 3E, normal cardiomyocytes had more dense and dispersive mitochondria compared with those in LPS group. After LPS stimulation, the mean area of mitochondria became shorter (Supplementary Figure 3A), and the proportion of cardiomyocytes with fragmented mitochondria was increased (Supplementary Figure 3B). Next, we analyzed ultrastructural changes by TEM. As shown in Figure 3F, normal cardiomyocytes had more large mitochondria with regular arrangement as compared with LPS group. Supplementation of IL-13 restored the number, size and arrangement of mitochondria in inflammatory cardiomyocytes. In addition, LPS-stimulated mitochondria were characterized by the presence of fragmented cristae and swollen Vacuoles.

### LPS Leads to Damage of Electronic Delivery Chain

**FIGURE 4:**
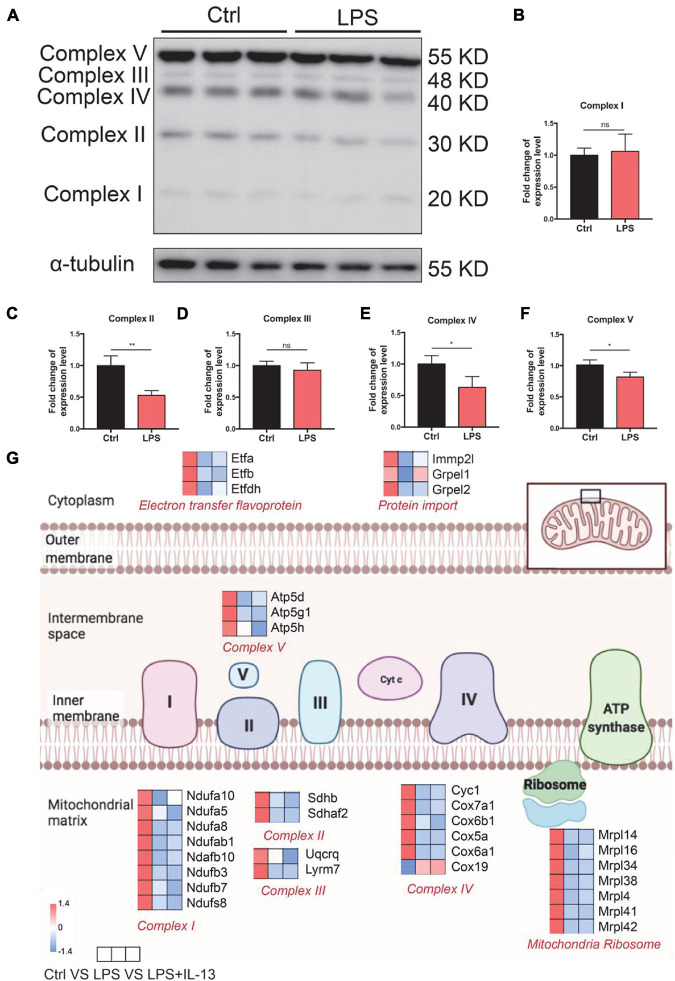
Change of electronic delivery chain in lipopolysaccharide (LPS)-induced cardiomyocytes. **(A)** Western immunoblotting and **(B–F)** quantification of multiple OxPhos proteins from primary cardiomyocytes induced by LPS (*n* = 3). **(G)** Expression level of representative genes related to electronic delivery chain by RNA-sequencing (RNA-seq) in the mouse heart. Data are presented as a heatmap (*n* = 6). Data are shown as the mean ± SEM. *P < 0.05; **P < 0.01; ***P < 0.001.

To investigate the cause of damaged ATP production, the expression level of OxPhos in control and LPS-induced cardiomyocytes was detected by Western Blot. It was found that the expression level of complex II, complex IV, and complex V was significantly decreased (Figures 4A–F). Furthermore, the hearts were harvested from the control and LPS-induced mice with or without IL-13 treatment for RNA-seq to explore the potential mechanisms. It was found that IL-13 treatment significantly up-regulated the expression of 62 genes and down-regulated the expression of 36 genes in the heart of LPS and IL-13 groups (*P* < 0.05 and | log_2_FC| > 1) (Supplementary Figure 4). Interestingly, of the genes significantly related to electronic delivery chain, only Grprl1 underwent significant change (Figure 4G), knowing that Grprl1 is responsible to transport proteins from the membrane to mitochondria matrix in an ATP-dependent manner. This finding suggests that IL-13 could not restore the electronic delivery chain activity at transcriptional level, and there may be other potential pathways.

### Interleukin-13 Reverts the LPS Induced Mitochondria Biogenesis Disorder

**FIGURE 5:**
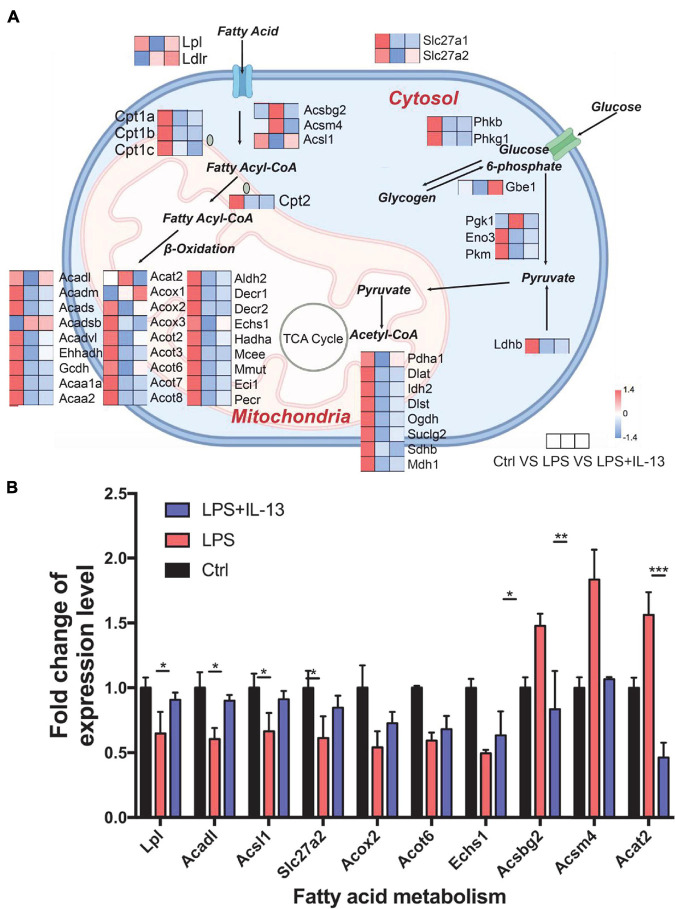
Change of mitochondria biogenesis in LPS-induced cardiomyocytes. **(A)** Expression level of representative genes related to mitochondria biogenesis by RNA-seq in the mouse heart. Data are presented as a heatmap (*n* = 6). **(B)** The expression level of genes related to fatty acid metabolism was validated by RT-qPCR (*n* = 3). Data are shown as the mean ± SEM. *P < 0.05; **P < 0.01; ***P < 0.001.

Knowing that IL-13 could not ameliorate LPS-induced mitochondria injury, we next analyzed the change of mitochondria biogenesis in LPS-induced cardiac injury. As shown in Figure 5A, totally 13 associated genes reverted after IL-13 treatment as compared with LPS group. Interestingly, among these genes, *Acadl*, *Acsl1*, *Acot6*, *Acat2*, *Acox2*, *Echs1*, *Acsm4*, *Acsbg2*, *Slc27a2*, and *Lpl* were responsible for fatty acid uptake and β-oxidation, knowing that they are the main energy source for the heart. Only three genes (Gbe1, Pdha1, and Prkaa1) were related to carbohydrate metabolism. Based on the results of heatmap analysis, we validated the fatty acid metabolism gene expression level in primary cardiomyocytes by RT-qPCR, and found that Lpl, Acadl, Acsl1, Slc27a2, Acsbg2, Acsm4, and Acat2 underwent significant changes in LPS + IL-13 group compared with LPS, which is in consistent with the result of RNA-seq (Figure 5B). These results suggest that IL-13 may alleviate the LPS induced mitochondria biogenesis disorder by increasing the expression level of fatty acid uptake and β-oxidation.

### Interleukin-13 Ameliorates Fatty Acid Uptake and Oxidation of Mitochondria

**FIGURE 6:**
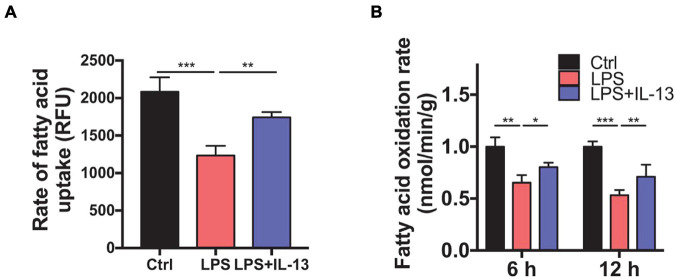
Interleukin-13 ameliorates fatty acid uptake and oxidation in mitochondria. **(A)** Rate of fatty acid uptake in primary cardiomyocytes. **(B)** Rate of fatty acid oxidation in primary cardiomyocytes. Data are shown as the mean ± SEM (*n* = 3). *P < 0.05; **P < 0.01; ***P < 0.001.

To determine whether IL-13 could ameliorate the fatty acid metabolism in LPS-stimulated primary cardiomyocytes, we detected the rate of fatty acid uptake and oxidation. As shown in Figure 6A, fatty acid uptake was impacted obviously by LPS compared with the control group, and IL-13 treatment improved the damaged uptake efficiency. In accordance with the results of uptake, IL-13 restored the activity of fatty acid oxidation at both 6 and 12 h after LPS stimulation (Figure 6B). These results suggest that IL-13 could effectively ameliorate sepsis-induced mitochondrial biogenesis dysfunction.

## Discussion

Sepsis-induced cardiac injury is one of the most common postoperative complications in the ICU, causing high morbidity and mortality. *SIC* is mainly characterized as systolic dysfunction in the serious inflammation environment. However, myocardial dysfunction in sepsis is a well-recognized but poorly understood condition without effective treatment.

Numerous mechanisms including calcium overload, ROS production, calcium overload, inactivation of ion channels and mitochondria dysfunction are involved in the development of *SIC*. Mitochondrial dysfunction is a hotspot of research on septic cardiomyopathy in recent years. Previous studies reported that LPS affected mitochondrial biosynthesis and eventually mediated the apoptosis of cardiomyocytes (Xin and Lu, 2020). Xu et al. (2018) demonstrated that several key regulators of mitochondria-associated apoptosis were abnormally expressed in the cecal ligation puncture (CLP) animal model, which may prove to be a promising potential target for the treatment of *SIC* with traditional Chinese medicine.

Sepsis-induced mitochondrial dysfunction is organ-specific and depends on the phase of the disease (Makrecka-Kuka et al., 2019). It is therefore significant to find a new regulator for mitochondrial dysfunction in the treatment of the disease. IL-13 is a key factor of type 2 immunity and plays an important role in fighting helminth infection, regulating asthma and tissue repair (Chu et al., 2021; Rodriguez-Rodriguez et al., 2021; Snodgrass et al., 2021). Previous research showed that IL-13 could maintain and increase the function and morphology of mitochondria in different diseases. For example, IL-13 could prevent and treat sepsis-induced brain dysfunction by enhancing the mitochondrial function and content in the brain microglia (Yan et al., 2020). In addition, latest research demonstrated that IL-13 mediated the mitochondrial metabolism from glycolysis switch into fatty acid oxidation, thus improving skeletal muscle endurance (Knudsen et al., 2020). These studies are consistent with the finding in our study that LPS induced obvious damage to mitochondrial function and morphology in terms of the decreased number, the smaller size, cell derangement and vacuolar formation in both *in vivo* and *in vitro SIC* models.

It is remarkable to note that β-oxidation of fatty acid, rather than glycolysis, is the primary energy supply for adult cardiomyocytes, which is different from other cells (Schulze et al., 2016; Cao et al., 2019). Recent studies reported that depressed cardiac function always accompanied with damaged mitochondrial fatty acid oxidation, and prevention of sepsis-induced fatty acid β-oxidation could improve heart function (Drosatos et al., 2011; Soraya et al., 2016). Our results also confirm that fatty acid oxidation plays a major role in the development of *SIC*. LPS mainly affects the transcription of genes involved in mitochondrial fatty acid uptake and β-oxidation, while IL-13 can restore the expression of these genes and rate of fatty acid uptake and oxidation to a certain extent, thereby reducing the apoptosis of cardiomyocytes caused by sepsis induced mitochondrial injury.

Clinically, *SIC* is a heterogeneous disease with different damage degrees of cardiac function and mitochondria caused by different sources of infection (Ehrman et al., 2018; Hollenberg and Singer, 2021). In this study, we used 10 mg/mL LPS to induce relatively consistent and stable endotoxemia, but different concentration gradients were missing (Ndongson-Dongmo et al., 2019; Vico et al., 2019). Another limitation of the current study is that *SIC* is a dynamic disease over time. In this study, we mainly focused on the myocardial protection in the early stage of the disease and set 6 h after LPS injection as the time point of observation. Pathophysiological changes and possible mechanisms of *SIC* with different severities and at different time points will be further evaluated in our subsequent experiments.

In conclusion, mitochondrial dysfunction is an important cause of *SIC* as represented by production of large amounts of ROS and myocardial cell apoptosis. IL-13 could effectively improve the ability of fatty acid uptake and oxidation in mitochondria, thereby reducing mitochondrial dysfunction and ameliorating apoptosis of *SIC*. Our results demonstrated that IL-13, working as an important immunity regulator, may prove to be an important potential target for the treatment of *SIC*.

## Data Availability Statement

The original contributions presented in the study are included in the article/Supplementary Material, further inquiries can be directed to the corresponding authors.

## Ethics Statement

The animal study was reviewed and approved by Ethics Committee of Shanghai Chest Hospital.

## Author Contributions

XG, TH, and SZ conducted the experiments. YW, HJ, and QM analyzed the data. KW and MZ wrote the manuscript. CW designed the manuscript. BH designed the methodology and supervised the study. All authors contributed to the article and approved the submitted version.

## Conflict of Interest

The authors declare that the research was conducted in the absence of any commercial or financial relationships that could be construed as a potential conflict of interest.

## Publisher’s Note

All claims expressed in this article are solely those of the authors and do not necessarily represent those of their affiliated organizations, or those of the publisher, the editors and the reviewers. Any product that may be evaluated in this article, or claim that may be made by its manufacturer, is not guaranteed or endorsed by the publisher.
