# Supplementary material for: IL-13 Alleviates Cardiomyocyte Apoptosis by Improving Fatty Acid Oxidation in Mitochondria
Source: Front Cell Dev Biol. 2021 Sep 17;9:736603. doi: 10.3389/fcell.2021.736603 (PMC8484794; doi:10.3389/fcell.2021.736603)
Supplement: Supplementary file 1 [file Data_Sheet_1.docx]

**Table S1**

**List of primer sequences used for RT-qPCR**

| **Gene** | **Forward sequence (5’-3’)** | **Reverse sequence (3’-5’)** |
| --- | --- | --- |
| *Rplp0* | AGATGCAGCAGATCCGCAT | AGATGCAGCAGATCCGCAT |
| *Lpl* | GCGTAGTTCCAGCAGCAAAG | AGAAATCTCTTCCCGCGTCTG |
| *Acadl* | ATGCAAGAGCTTCCACAGGAAA | CAGAAATCGCCAACTCAGCAAT |
| *Acsl1* | GGCCGCGACTCCTTAAATAGC | CTCTATGCAGAATTCTCCTCCGC |
| *Slc27a2* | GGAACCACAGGTCTTCCAAA | TAAAGTAGCCCCAACCACGA |
| *Acox2* | CATCCAACGTGACCCAGTGTT | AAATGCGTTCAGGACCGTCTT |
| *Acot6* | AGTATCGGTGGCTTATGTGAACA | AGGCGAACGTCACTCAGATTT |
| *Echs1* | CCCAGAACTACGGCGCTTC | CCACGCTGCTATTCTTTCCTT |
| *Acsbg2* | GAAGGCGGAAGACCTTGAGAG | AACCTCAGGAGCACTTCTCCA |
| *Acsm4* | ATTCTGCCCCGTATCCCTGAA | TTCCTGGCATGAATACAAGCC |
| *Acat2* | CCCGTGGTCATCGTCTCAG | GGACAGGGCACCATTGAAGG |

**Figure S1**

**
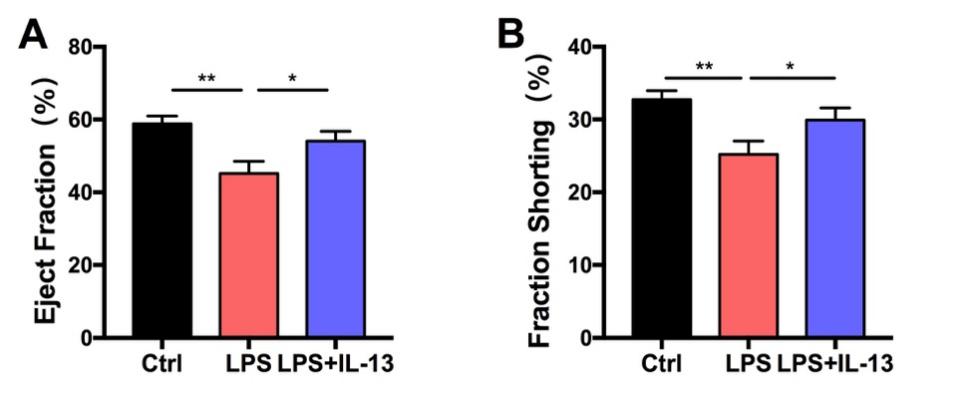
**

**Figure S1:** Quantified (A) Eject fraction (EF%) and (B)Fraction shorting of Echocardiography (ECG)

**Figure S2**


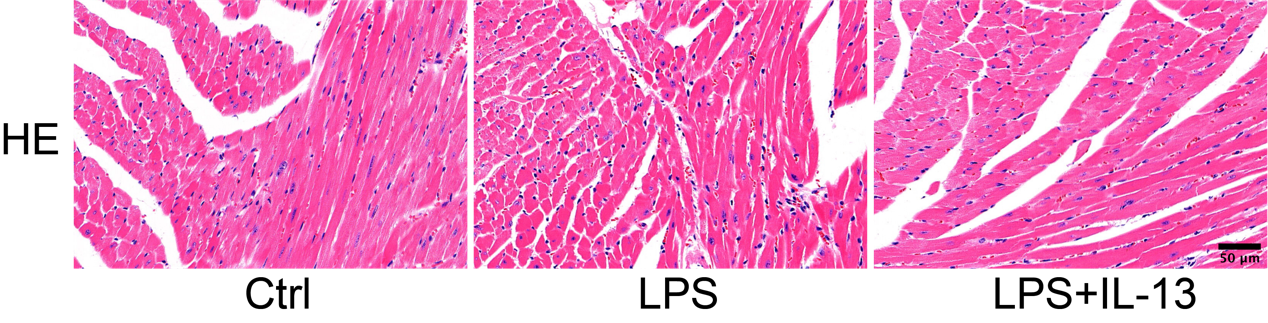


**Figure S2**: Representative pictures of H&E Staining.

**Figure 3**


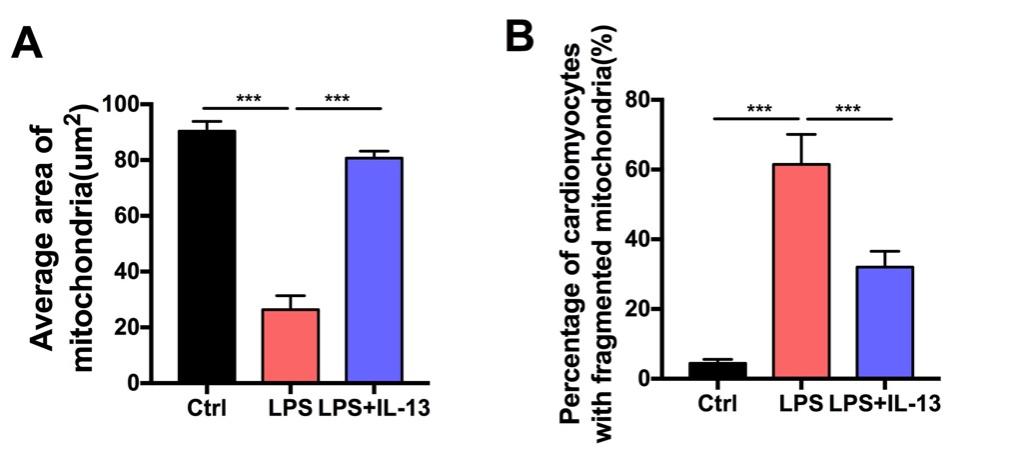


**Figure 3:** Quantified results of average area of mitochondria (A) and percentage of cardiomyocytes with fragmented mitochondria(B).

**Figure S4**


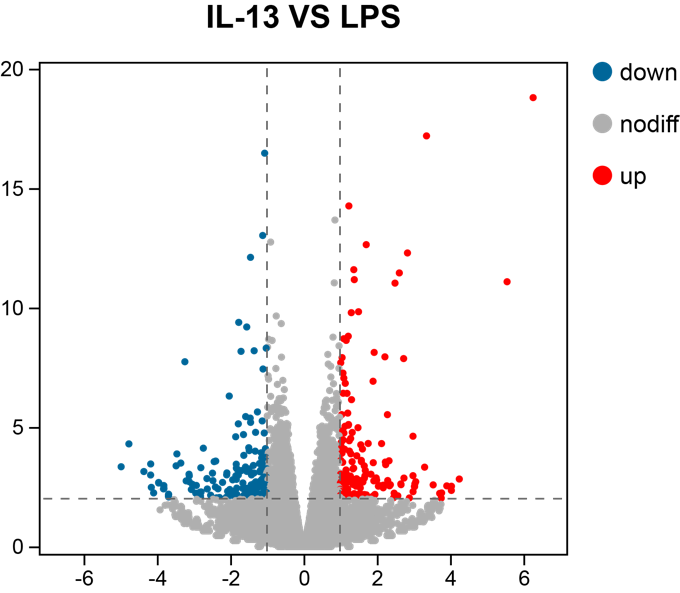


**Figure S4:** Volcano picture of differential expressed genes (DEGs) of IL-13 group versus LPS group. P<0.05, |Log2FC|>1.
